# 3D Interconnected Binder-Free Electrospun MnO@C Nanofibers for Supercapacitor Devices

**DOI:** 10.1038/s41598-018-26370-z

**Published:** 2018-05-22

**Authors:** Mohamed Ramadan, Ahmed M. Abdellah, Saad G. Mohamed, Nageh K. Allam

**Affiliations:** Energy Materials Laboratory (EML), School of Sciences and Engineering, The American University in Cairo, New Cairo, 11835 Egypt

## Abstract

Rational design of binder-free materials with high cyclic stability and high conductivity is a great need for high performance supercapacitors. We demonstrate a facile one-step synthesis method of binder-free MnO@C nanofibers as electrodes for supercapacitor applications. The topology of the fabricated nanofibers was investigated using FESEM and HRTEM. The X-ray photoelectron spectroscopy (XPS) and the X-ray diffraction (XRD) analyses confirm the formation of the MnO structure. The electrospun MnO@C electrodes achieve high specific capacitance of 578 F/g at 1 A/g with an outstanding cycling performance. The electrodes also show 127% capacity increasing after 3000 cycles. An asymmetric supercapacitor composed of activated carbon as the negative electrode and MnO@C as the positive electrode shows an ultrahigh energy density of 35.5 Wh/kg with a power density of 1000 W/kg. The device shows a superior columbic efficiency, cycle life, and capacity retention.

## Introduction

The development of energy storage devices is indisputably one of the great challenges in the 21^st^ century to meet the needs of modern society^1,2^. To this end, supercapacitors (SCs), also known as ultracapacitors or electrochemical capacitors, have gained much attention as the next generation power storage devices, mainly due to their safe operation, outstanding cycling life, higher energy than conventional capacitors, and higher power density than batteries and fuel cells^3,4^. However, the bottle nick preventing their efficient use in future applications is their comparatively low energy density^5–7^. In this regard, transition metal oxide pseudocapacitive materials are primarily being used in SCs devices due to their high capacitance compared to carbonaceous materials^8,9^. While the former stores charge via rapid and reversible faradaic reactions at the electrode surface, capable of storing higher energy, the latter stores energy physically via the formation of electric double layer with lower energy storage but higher cycle life stability^10–13^. Among the various oxides used^14–19^, manganese oxides (Mn_x_O_y_) have been widely investigated as promising candidates for SCs^20–23^. Recently, manganese monoxide (MnO) has attracted the attention as an active material for supercapacitor applications owing to its abundance, low cost, environmental compatibility, and high theoretical specific capacitance (1360 F g^−1^) that is higher than that of MnO_2_ (1110 F g^−1^) counterpart^24–26^. However, MnO suffers from limited cyclic stability, poor conductivity and loss of surface active sites within the synthesis process due to the agglomeration of the particles^27,28^. Consequently, the reported specific capacitance is still far from the theoretical specific capacitance. To this end, different approaches have been utilized including nanostructuring the material and/or mixing with conductive materials. Hu *et al*. reported the fabrication of MnO_2_ nanoflowers supported by a backbone of nanoporous gold (NPG) wires, which enabled fast ionic/electronic transfer at the electrode surface^29^. Yu *et al*. reported the hydrothermal growth of MnO@C directly on carbon cloth and its use as a negative binder-free electrode^23^. Although carbon coating provides efficient electron transfer pathway, the capacitance dropped to 86% after 5000 cycles^30^. Moreover, Liao *et al*. have synthesized MnO nanoparticles anchored to vertically aligned graphene sheets (VAGN), showing 80% capacitance retention after 4000 cycles^27^. Otherwise, Wang *et al*. reported that MnO@mesoporous carbon achieved a high cycling stability of 110% of its original value^24^. However, the electrode material showed low specific capacitance (160 F @ 1 A g^−1^)^31^. To this end, the fabrication of MnO-based materials with integrated high properties (High cycling stability, high specific capacitance, facile and scalable synthesis methods and facile processing) is still an open challenge.

Herein, we demonstrate the ability to grow MnO nanoparticles within interconnected carbon matrix via direct electrospinning on graphite substrate followed by thermal treatment directly in one step without incorporation of pre-synthesized manganese oxide nanostructures with polymers^32,33^. The resulted binder-free electrode was used as a positive electrode in asymmetric supercapacitor devices. The fabricated composite material system was chosen for the following reasons: (1) carbon matrix not only does enhance the electronic/ionic transfer pathways^34^, but also provides a robust support for MnO nanoparticles, preventing the agglomeration during the synthesis and utilization^28^, (2) the binder-free electrode avoids the contact impedance between the current collector and the active film, which simplifies electrode fabrication steps^35^, allowing for a full utilization of the composite material, and (3) the 3D interconnected matrix provides a high capacity and cycling stability^36^.

## Experimental section

### Synthesis of MnO@C

All chemicals were directly used as purchased without further purification. 10% Polyvinylpyrrolidone (PVP Mw = 1300000) solution was prepared in ethanol (99.9%). Then, 2 M manganese chloride solution was slowly added to the solution of PVP with constant stirring for 6 hours. The spinning solution was then transferred into a syringe with fine capillary metallic needle. The electrospinning process was carried out using a conventional electrospinning setup (MECC Nanon-01A, Japan) at a working distance of 15 cm, a working voltage of 15 kV, and a feeding rate of 0.6 ml/h. Samples were collected on graphite sheets and aluminum foils. The dried nanofibers were annealed for 4 h at 650 °C with 1 °C/min uprate in argon gas. For comparison, a pure graphite sheet was annealed under the same conditions and weighed before and after annealing. As no mass loss was observed, the mass of the formed MnO@C was measured by the same way.

### Materials characterization

The microstructure and morphology of the samples were observed using field emission scanning electron microscopy (FESEM, Zeiss SEM Ultra 60, 5 kV) and high-resolution transmission electron microscope (HR-TEM, JOEL JEM-2100) operating at 200 kV accelerating voltage of. X-ray powder diffraction (PXRD) patterns were recorded on a Panalytical X′pert PRO MPD X-ray Diffractometer with Cu Kα radiation (λ = 0.15418 nm, 45 kV, 40 mA). The elemental composition was investigated using X-ray photoelectron spectroscopy (XPS, ESCALAB 250Xi, Thermo Scientific).

### Electrochemical measurements

The three-electrode configuration was adopted to measure the electrochemical behavior of the electrode material. The formed electrode (1 × 2 cm^2^) acted as the working electrode, a platinum foil served as the counter electrode, and Ag/AgCl electrode as a reference electrode and 1 M Na_2_SO_4_ as the electrolyte. Cyclic voltammetry (CV) and electrochemical impedance spectroscopy (EIS) were carried out using a BioLogic SP-200 potentiostat. Constant current charge/discharge (CCCD) measurements were conducted using a CHI model 700D series electrochemical workstation. Specific capacitance was calculated based on the mass of the MnO@carbon.

## Results and Discussion

**Figure 1:**
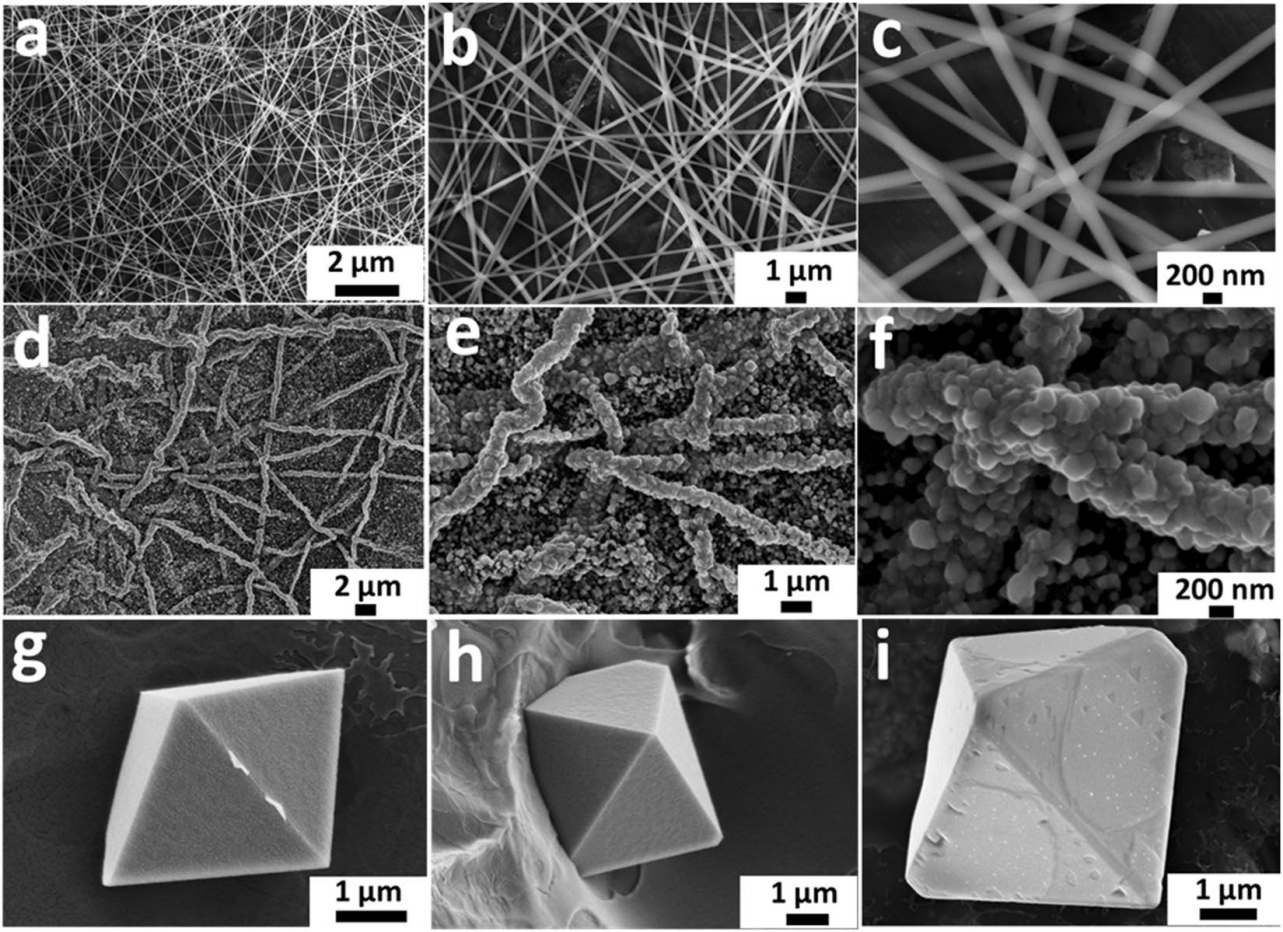
(**a**–**c**) As-spun NFs, (**d**–**f**) MnO@C collected on graphite substrate at 650 °C for 4 h, and (**g**–**i**) MnO without graphite substrate at 650 °C for 4 h.

Figure 1a–c shows field emission scanning electron microscope (FESEM) images of the as-spun nanofibers (NFs), indicating the successful formation of smooth, tangled and homogeneous fibers with lengths exceeding 10 µm. Figure 1d–f shows the NFs after annealing at 650 °C for 4 hours under argon stream and collected onto graphite sheets. Note that the MnO nanoparticles were grown along the nanofibers forming an interconnected dense matrix. Note also that the particle size is less than 100 nm when collected directly into graphite substrate. However, MnO grew in unfavorable large and separate crystals up to 2 µm when annealed without the use of graphite substrate forming free fibers; see Fig. 1g–i.

**Figure 2:**
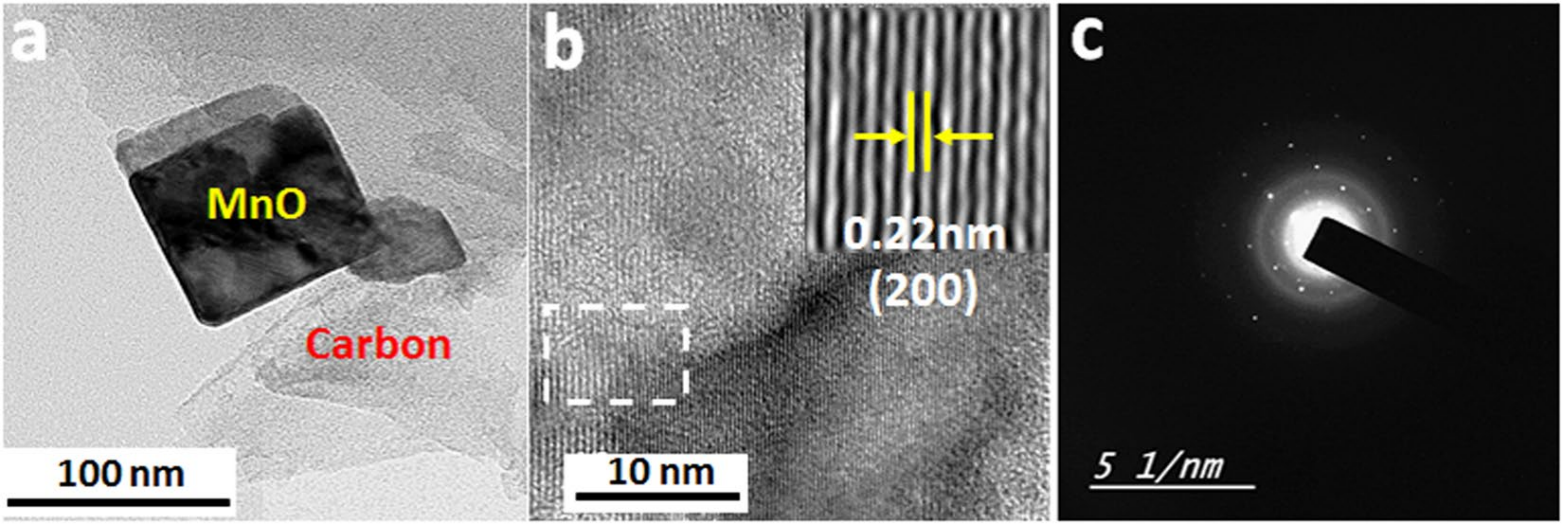
(**a**) TEM, (**b**) HRTEM, and (**c**) SAED images of MnO@C.

Accordingly, graphite substrate has provided a suitable surface for the growth of MnO nanoparticles, preventing their agglomeration during nucleation and helping in the formation of interconnected 3D matrix of MnO@C. Furthermore, the microstructure of MnO nanoparticles was investigated by transmission electron microscope (TEM), as shown in Fig. 2a, where MnO octahedron nanoparticles possess a uniform tetragonal projected shape, which is consistent with the octahedron morphology seen by SEM. Note the carbon layer surrounding the MnO crystal, which is favorable for good electronic and ionic conductivity. Figure 2b reveals that the lattice fringes have a distinct interplanar spacing of 0.22 nm, in good agreement with (200) plane of the cubic MnO phase^37^. Further, the selected area electron diffraction (SAED) analysis (Fig. 2c) demonstrates that the as-synthesized MnO@C NFs have a high quality single-crystalline nature.

**Figure 3:**
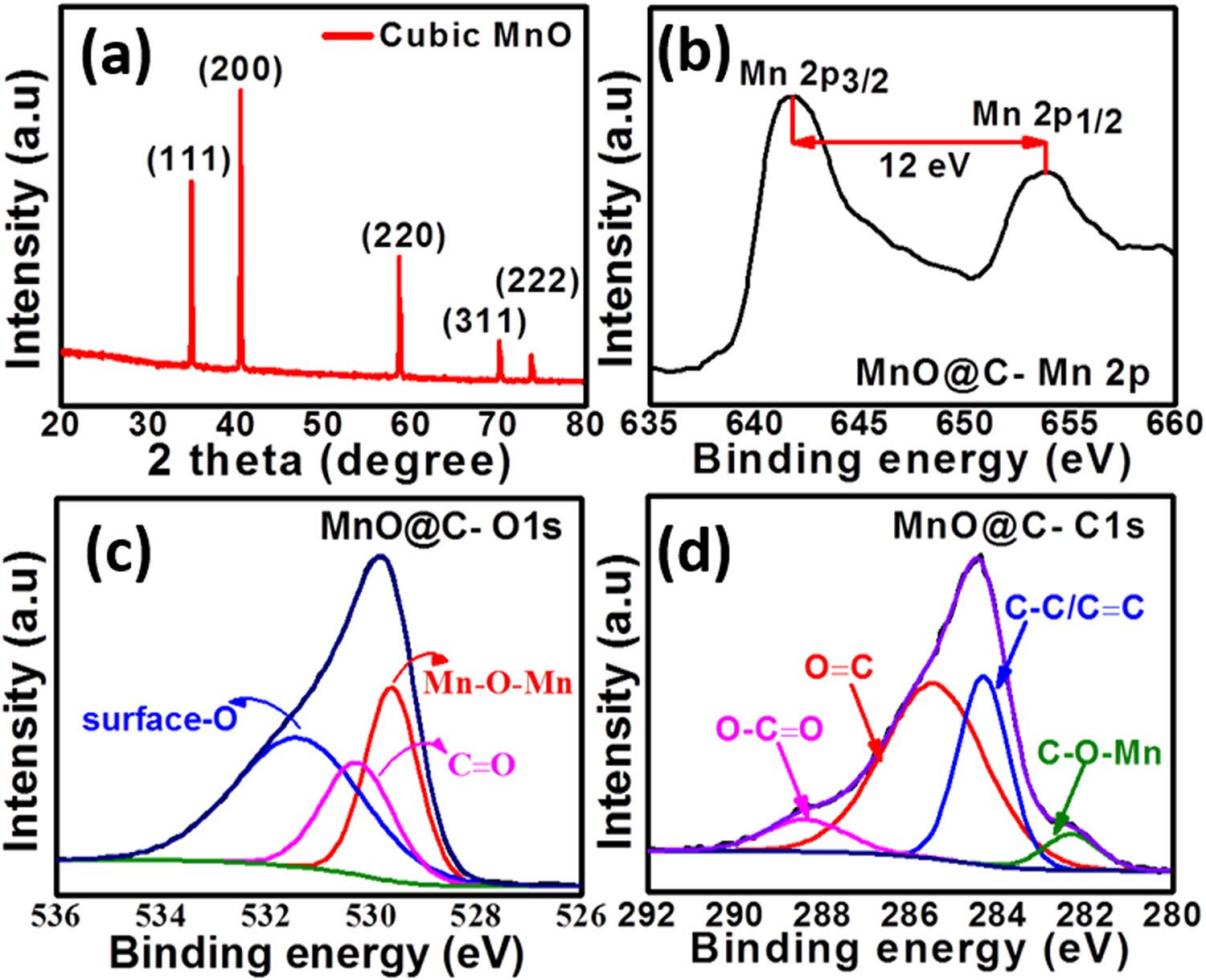
(**a**) XRD spectrum of the C/MnO, and XPS spectra for the (**b**) Mn2p, (**c**) O 1 s, and (**d**) C1s.

Figure 3a displays the XRD spectrum of the fabricated MnO nanoparticles. All the observed diffraction peaks is corresponded to Fm3m space group of the cubic MnO crystal (JCPDS No. 07-0230), in agreement with the TEM results^38^. No other peaks of impurities are detected, suggesting the successful synthesis of very pure composite electrodes with a manifested crystallinity as confirmed via SAED and HRTEM results. To further investigate the surface and chemical composition of the fabricated electrode materials, XPS analysis was performed. The high-resolution Mn 2p spectrum (Fig. 3b) exhibits two signals at 641.8 eV for Mn 2p_3/2_ and 653.8 eV for Mn 2p_1/2_, which are characteristic of MnO, in agreement with those reported in literature^39–41^. This was also confirmed via the spin orbit splitting of 12 eV of MnO.41 The XPS peak of the O1s peak (Fig. 3c) can be deconvoluted into three peaks at 529.6, 530.3 and 531.4 eV, which correspond to Mn-O-Mn, C=O and surface-O bonds, respectively. Furthermore, the deconvolution of C 1 s peak (Fig. 3d) shows four types of bonds within MnO@C matrix, including C-O-Mn (282.3 eV), C-C/C=C (284.3 eV), C-O (285.5 eV) and O-C=O (288.4 eV). The XPS data confirmed that MnO nanoparticles were successfully bonded to carbon during crystal growth, which is in agreement with the morphological observations showed by SEM and TEM analyses.

**Figure 4:**
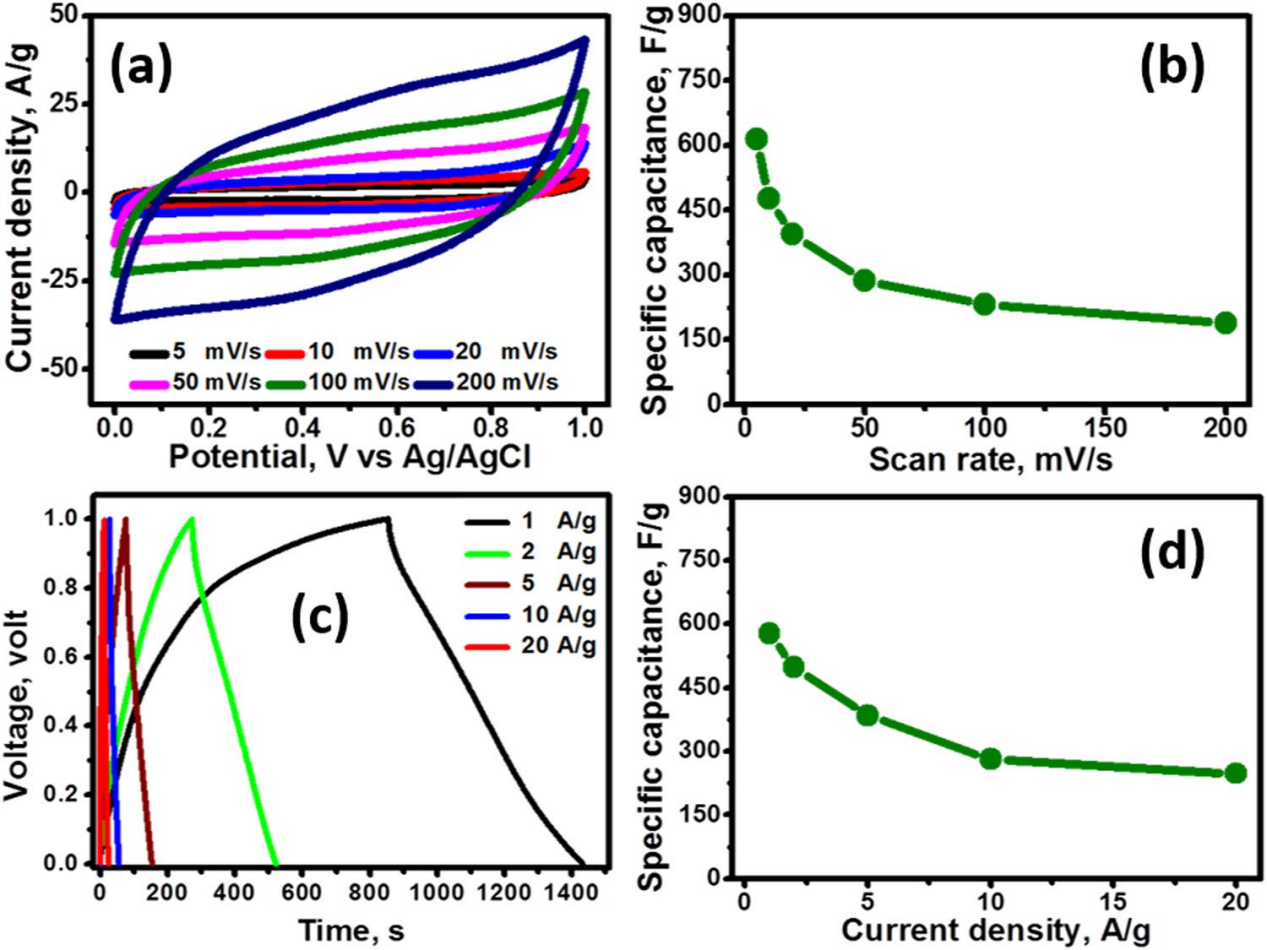
(**a**) CV curves of MnO@C at different scan rates, (**b**) Csp versus scan rates, (**c**) constant current charge/discharge curves at different current densities from 1 to 20 A g^−1^, and (**d**) Csp versus discharge current densities.

The electrochemical characteristics of MnO@C were assessed by cyclic voltammetry (CV), constant current charge and discharge (CCCD) and potentioelectrochemical impedance spectroscopy (PEIS) in a 3-electrode electrochemical cell containing 1 M Na_2_SO_4_. Figure 4a displays the cyclic voltammograms of MnO@C electrodes over a potential window of zero to 1 Volt (V) vs. Ag/AgCl at various scan rates (5 to 200 mV s^−1^). The CV plots at low scan rates (<50 mV s^−1^) appeared as approximately quasi rectangular shape without any battery-like redox peaks, indicating that the cell potential is varying linearly with time. Besides that, the cyclic voltammograms showed symmetric characteristics with respect to voltage axis at zero current. All these observations reflect that MnO@C possess a close-to-ideal pseudocapacitive electrochemical characteristics^42^. On the other hand, at high scan rates (50–200 mV/s), the CV plots reveal a little deviation from rectangular style owing to the electrode polarization, which can be due to the diffusion difficulty which faced Na^+^ ions to get intercalated deeper within the bulk of the material at high scan rates^43^. The charge mechanism at low/high scan rates is based on the intercalation/deintercalation and adsorption/desorption encompassing bulk and surface similar to the other manganese oxides reported in literature^44,45^. This behavior is accompanied by oxidation/reduction between Mn^+2^ and Mn^+3^ ions at the bulk and surface the of the material^27^. Figure 4b reveals the variation of the specific capacitance (Csp, CV, F g^−1^) of MnO@C with the scan rate calculated from the CV plots according to Eq. 1:

1 $${{\rm{C}}}_{{\rm{s}}}=\frac{{\int }^{}I\,\text{dV}}{{\rm{Vm}}\,{\rm{\Delta }}V}$$

where Cs is the specific capacitance, I is the response current density, v is the potential scan rate, ∆V is the potential window, and m is the mass of electrode material. The specific capacitance of the electrode material (normalized to the mass of MnO@C) reached 615 F g^−1^ at a scan rate of 5 mV s^−1^ and drops to 199.8 F g^−1^ at a scan rate of 200 mV s^−1^. Additionally, CCCD curves measured at various current densities (1 to 20 A g^−1^) are illustrated in Fig. 4c. The nearly symmetric charge and discharge further validates the superior pseudocapacitance behavior of the electrode material, in agreement with the CV results. Based on the CCCD results, the specific capacitance (Csp) can be calculated from each discharging curve according to Eq. 2:

2 $${{\rm{C}}}_{{\rm{sp}}}=\frac{{\rm{I}}\,\text{dt}}{{\rm{m}}\,\text{dV}}$$

where *dt* is the discharging time (s), I is the discharging current (A), m is the mass of the active material (g) within the electrode and dV is the discharging potential range (V). Figure 4d shows the variation of the Csp with the discharge current density. The calculated Csp values obtained from each discharging curve are 578, 497.4, 385, 281 and 248 F.g^−1^ at 1, 2, 5, 10 and 20 A g^−1^, respectively. Note the gradual decrease in the specific capacitance with increasing the current density, as the mass efficiency/utilization gets decreased at high current densities. However, MnO@C electrode was capable of maintaining about 42.9% of its initial specific capacitance as the current density was increased from 1 A g^−1^ to 20 A g^−1^. Note also that the specific capacitances calculated from both CV and CCCD have very close values. Thus, CV and CCCD methods effectively determine the capacitance of the electrode.

**Figure 5:**
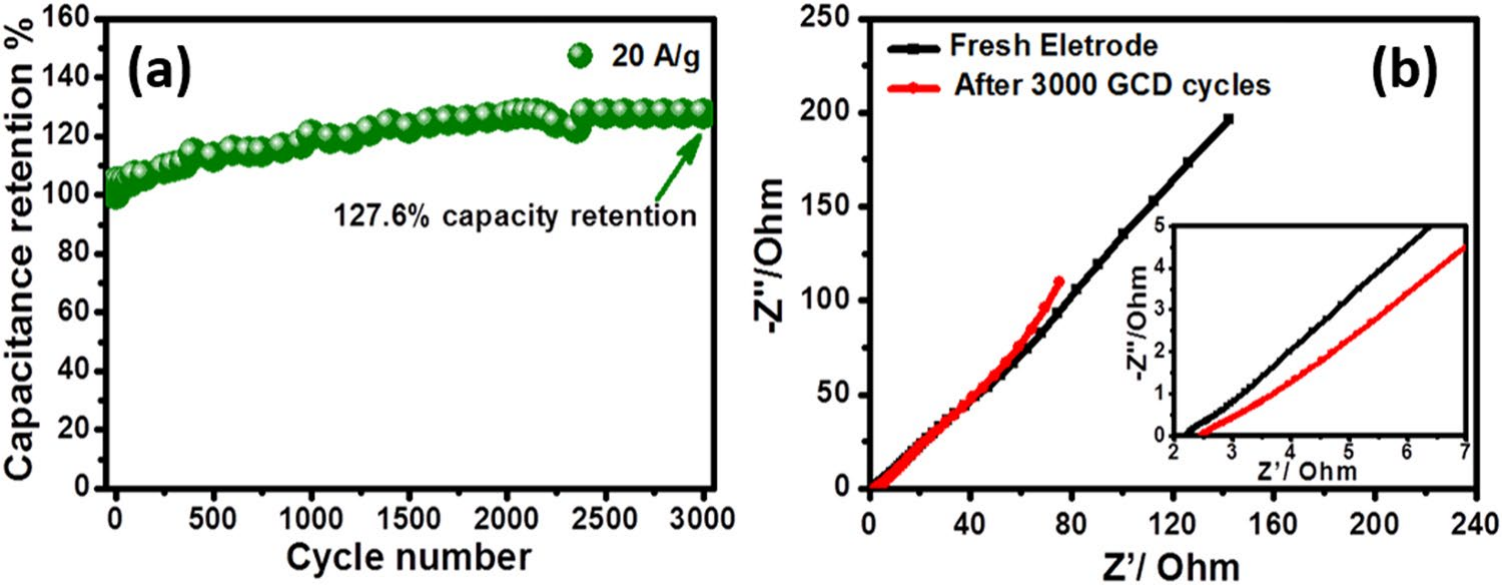
(**a**) Cycling stability of MnO@C at 20 A g^−1^ and (**b**) Nyquist plot of MnO@C before and after CCCD stability cycles (inset is the magnification at high frequencies).

Moreover, as the cycle life is a unique metric parameter of supercapacitors in practical application, the cycling performance of the fabricated MnO@C electrodes was investigated at high current density (20 A g^−1^), Fig. 5a. The electrodes retained excellent capacitance retention with 127% capacity increase after 3000 cycles. These results reveal the superiority of MnO@C electrode over many reported Mn_x_O_y_ electrodes^43,46,47^. Upon cycling, the surface wettability increases, which facilitates the continuous diffusion of the electrolyte ions into the electrode microstructure with better pathways reaching more deep particles. This diffusion enhancement leads to an activation effect throughout the electrochemical cycling, resulting in capacitance increase during cycling^31,48^. This also could be attributed to the superior architecture design of the electrode material, which provides a conductive matrix (carbon) throughout the MnO particles with smooth electron and ion transport pathways. Without this supporting design, the electrode material would suffer from low cycling stability. This issue can also be corroborative by determining the equivalent series resistance (ESR) of the electrode material as well as the charge transfer resistance. On the other hand, PEIS measurements were executed for the MnO@C electrode material before and after 3000 cycles in the frequency range 100 kHz–10 mHz with an excitation signal of 5 mV.

No semicircles were detected at high frequency (Fig. 5b inset), indicating no/minimal charge transfer resistance with fast electrode kinetics even after 3000 cycle, revealing the importance of binder-free electrode design to increase the charge transfer at the interface of active material/current collector^49,50^. Besides, the ESR slightly increases (from 2.22 to 2.47Ω), indicating a negligible Ohmic loss during cycling. Furthermore, the near-vertical line in the low frequency range alludes to the good capacitive behavior of the electrode material with low electrolyte diffusion impedance and fast electron transfer within the electrode material. Moreover, the observed straight line in the low frequency range became a little steeper after 3000 cycle, indicating easier diffusion, in agreement with the capacitance increase during the cycling stability test^51^.

To further assess the MnO@C electrode for real applications, a two-electrode asymmetric supercapacitor device was assembled by integrating activated carbon as the negative electrode, MnO@C electrode as the positive electrode, and a filter paper as the separator in 1 M Na_2_SO_4_ aqueous electrolyte. Besides, it is well-known that the voltage in an asymmetric capacitor will divide relying on the capacity of each electrode. Consequently, the optimum cell voltage with maximum capacitance and best cycle life would be achieved if the charges at both positive/negative (q+/q−) electrodes are balanced. As the stored charges (q+/−) are relevant to the potential window (ΔE), the specific capacitance (Cs), and the mass (m) of the electrode are relevant according to Eq. 3:

3 $${\rm{q}}={\rm{\Delta }}E\times {\rm{Cs}}\times {\rm{m}}$$

Hence, the masses of the negative and positive electrodes were balanced before assembling the asymmetric supercapacitor according to Eq. 4^52–54^

4 $$\frac{{{\rm{m}}}_{+}}{{{\rm{m}}}_{-}}=\frac{{{\rm{C}}}_{{\rm{s}}}^{-}{{\rm{\Delta }}V}^{-}}{{{\rm{C}}}_{{\rm{s}}}^{+}{{\rm{\Delta }}V}^{+}}$$

**Figure 6:**
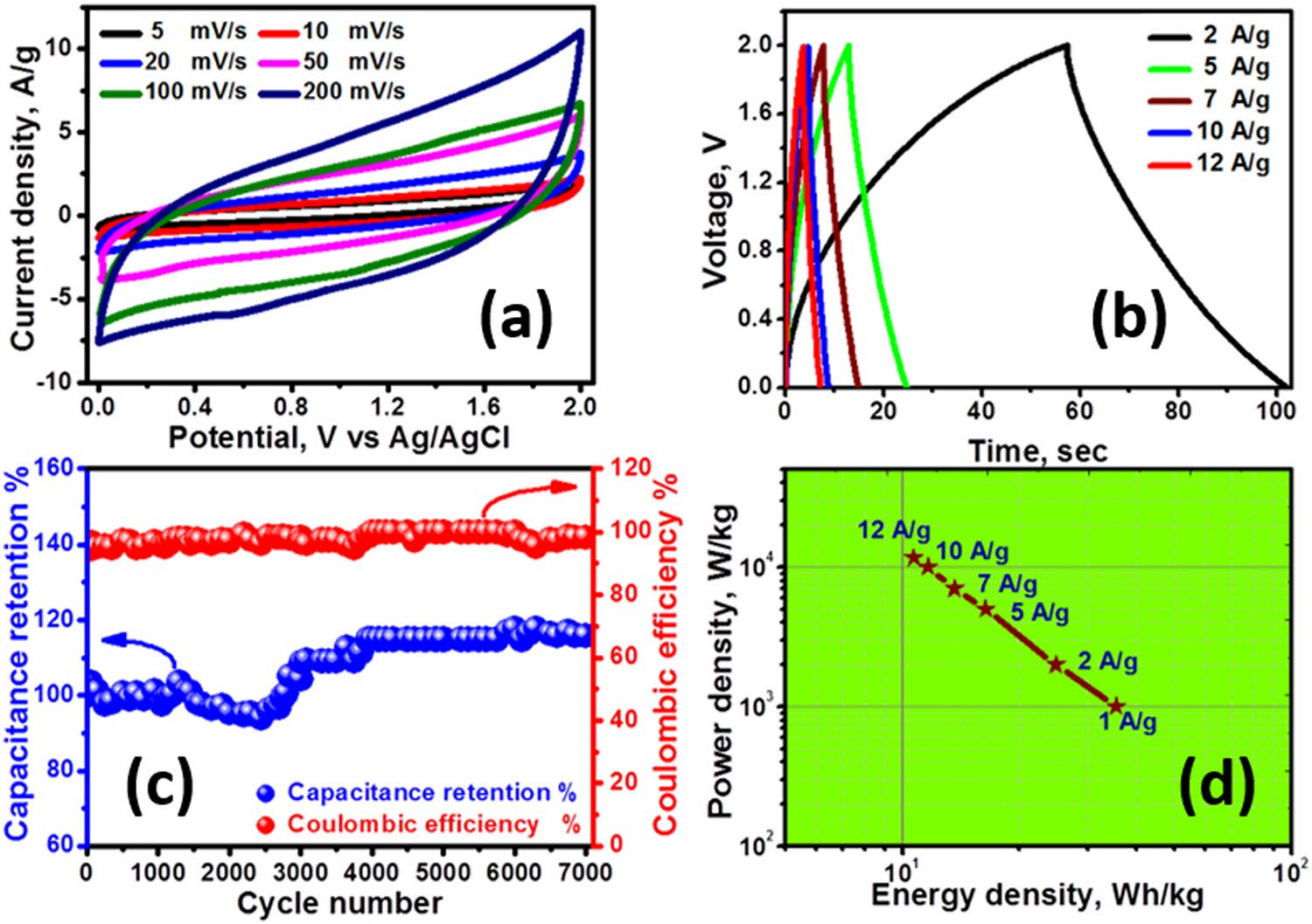
(**a**)CV curves of MnO@C//AC device at various scan rates, (**b**) CCCD curves of the asymmetric device at different current densities, (**c**) capacitance retention and columbic efficiency over 7000 CCCD cycles, and (**d**) Ragone plot of the device.

Figure 6a shows the CV curves of the asymmetric MnO@C//AC supercapacitor at different scan rates. The capacitor reveals an outstanding pseudocapacitive/capacitive behavior with a quasi-rectangular CV diagrams, which deviates a little at high scan rates (<50 mV s^−1^). It is also noticed that the device achieved a maximum operating cell voltage of 2 V. Thus, it would be a positive sign to obtain a high energy density supercapacitor in aqueous medium. The capacitive property of the assembled MnO@C//AC device was further investigated via CCCD tests at different current densities from 2 to 12 A g^−1^ in a potential window of 0–2 V. Figure 6b shows that all of the CCCD curves generally reveal a nearly triangle shape. This obvious symmetry of the curves suggests that the device works with good columbic efficiency and excellent electrochemical reversibility.

The stability test of the assembled MnO@C//AC device was performed at a discharging current density of 12 A g^−1^ for 7000 cycles using the CCCD technique. Figure 6c shows that the capacitance gradually decreases during the first 2500 cycles compared to the first cycle. Then, the electrode retrieved its capacitance with stepwise increase till capacitance retention of ~115% was achieved at 4000th cycle. After that, the capacitance gradually increased to 115.7% of its initial value even after 7000 cycles. Besides this superior stability, the fabricated MnO@C//AC supercapacitor showed very high columbic efficiency, remaining about 100% during the 7000 cycles.

Power (P) and energy (E) densities are considered the two substantial performance metrics of supercapacitor devices. Figure 6d shows the Ragone plot, which relates the energy density to power density of the MnO@C//AC supercapacitor at various charge-discharge current densities. Note that E&P were calculated from the CCCD graphs according to Eqs 5 and 6:

5 $${\rm{E}}=[{\rm{Cs}}({\rm{\Delta }}V)2]/2$$

6 $${\rm{P}}={\rm{E}}/{\rm{\Delta }}{\rm{t}}$$

where E, Cs, ∆V, P, and ∆t are the energy density, specific capacitance, potential window, power density and discharge time, respectively. Our supercapacitor can deliver an ultrahigh energy density of 35.5 Wh/kg at 1 A/g with a power density of 1000 W/kg. Upon increasing the current density to 12 A/g, in order to utilize the device at higher power density, the power density increased to 11666.6 W/kg while its energy density was maintained at ~10.7 Wh/kg.

## Conclusion

We demonstrate the successful fabrication of MnO@C via electrospinning method. The XRD, XPS and TEM results confirmed the formation of 3D interconnected MnO@C with high crystallinity and purity. The binder-free MnO@C electrodes showed very high specific capacitance (578 Fg^−1^) in 1 M Na_2_SO_4_ at 1 Ag^−1^ with excellent cycling stability. MnO@C was examined as the positive electrode in asymmetric device; delivering a high energy density of 35.5 Wh kg^−1^ with a power density of 1000 W kg^−1^ with very high columbic efficiency and cycling stability.
